# A fuzzy co-clustering algorithm for biomedical data

**DOI:** 10.1371/journal.pone.0176536

**Published:** 2017-04-26

**Authors:** Yongli Liu, Shuai Wu, Zhizhong Liu, Hao Chao

**Affiliations:** School of Computer Science and Technology, Henan Polytechnic University, Jiaozuo, Henan, China; Jiangnan University, CHINA

## Abstract

Fuzzy co-clustering extends co-clustering by assigning membership functions to both the objects and the features, and is helpful to improve clustering accurarcy of biomedical data. In this paper, we introduce a new fuzzy co-clustering algorithm based on information bottleneck named ibFCC. The ibFCC formulates an objective function which includes a distance function that employs information bottleneck theory to measure the distance between feature data point and the feature cluster centroid. Many experiments were conducted on five biomedical datasets, and the ibFCC was compared with such prominent fuzzy (co-)clustering algorithms as FCM, FCCM, RFCC and FCCI. Experimental results showed that ibFCC could yield high quality clusters and was better than all these methods in terms of accuracy.

## Introduction

Nowadays, the amount of biomedical data grows rapidly, which makes it difficult for medical workers and patients to find the information they need. The clustering technique can identify the latent structure and knowledge behind large-scale biomedical data, and therefore play an important role in reorganizing biomedical data and helping users find relevant information. This technique tries to generate a set of clusters where intra-cluster similarity is maximized and inter-cluster similarity is minimized, and is widely used for such applications as automatic categorization of text, grouping gene expression data, and others [1,2].

In recent years many researchers have studied data mining and presented a number of clustering algorithms [3–7]. These algorithms can be divided into hard and soft clustering algorithms [8]. Hard clustering has been studied extensively and well accepted by the scientific community. For example, Chen et al [9] studied hard clustering and proposed an automated two-level variable weighting clustering algorithm for multiview data, which can simultaneously compute weights for views and individual variables. In hard clustering, each object belongs to exactly one cluster, while soft clustering allows an object to belong to more than one cluster. For example, nodular goiter can be put into two clusters, Thyroid Surgery and Endocrinology. As another example, the atypical hyperplasia could be considered as normal endometrium or abnormal endometrium by different doctors. Above examples tell us soft clustering may be more reasonable than hard clustering, because many times we cannot put an object into just one cluster.

When mentioning soft clustering, we need to talk about fuzzy clustering, which is regarded as the combination of clustering and fuzzy sets. Fuzzy clustering is relatively new. Its representative algorithm is the Fuzzy *c*-Means (FCM) algorithm, which is the fuzzy version of traditional K-Means clustering algorithm. The main difference is that the K-Means is a hard algorithm, while the FCM is a soft algorithm. In other words, K-Means represents the affiliation of objects to clusters by memberships taking values 0 and 1, however, in FCM, the memberships take values in the real unit interval [0, 1] [10,11]. Therefore, the FCM is, indeed, the fuzzy version of the K-Means. Conversely, the K-Means can be regarded as a special case of the FCM. Researchers have developed FCM in recent years. Jiang et al [12] studied how to combine the clustering result from each view and proposed a collaborative fuzzy c-means (Co-FCM) algorithm.

The FCM is a kind of one-dimensional clustering algorithm. That is to say, when grouping a disease-symptom contingence table, the FCM assumes that there is no relationship between the symptoms, and just classifies the diseases based on the symptoms. Actually, we are aware that there may exist mutual influence between some diseases, for example, there is a close relation between increased pulse pressure and types of metabolic diseases. As this is the case, it is unscientific to neglect the correlations between the symptoms. If the disease-symptom contingence table is considered unrepresentative, we can discuss a more typical example, i.e. a document-word matrix. In exactly the same way, if we analyze a document-word matrix, we had better think highly of the correlations between words, because as is known to all, some words are synonyms and some words are antonyms. Thus it can be seen, when we are analyzing an object-feature contingence table for clustering, we should group both the object and feature dimensions. Accordingly, the two-dimensional fuzzy clustering algorithms, called fuzzy co-clustering algorithms, are better than the one-dimensional FCM, especially when there are strong correlations between features.

Fuzzy co-clustering can simultaneously group objects and features based on the co-occurrence information [13–15]. As a result, more relationships between objects and features are kept, and therefore we can get more interpretable clustering results. At the same time, because the features are also partitioned into feature clusters, which means the feature dimensionality is reduced significantly, the clustering process will be accelerated. So far, many fuzzy co-clustering algorithms have been presented. The FCCM (Fuzzy Clustering for Categorical Multivariate data) [14] is the best-known fuzzy co-clustering algorithm, which can be regarded as a two-dimensional FCM. Other prominent fuzzy co-clustering algorithms include FCR (Fuzzy co-Clustering with Ruspini’s condition) [16], FCCI (Fuzzy Co-Clustering algorithm for Images) [17], PFCC (Possibilistic Fuzzy Co-Clustering) [18], RFCC (Robust Fuzzy Co-Clustering) [19] and SS-HFCR (Heuristic Semi-Supervised Fuzzy co-Clustering algorithm)[20], etc. In order to compare these algorithms, we first give the explanations on the mathematical notations used in this paper (as **Table 1**). With the mathematical notations, objective functions of some popular fuzzy co-clustering algorithms mentioned above are provided in **Table 2**.

**Table 1:** List of mathematical notations.

Notation	Description
***C*, *N*, *K***	Numbers of (co-)clusters, objects and features
***u***_***ci***_	Fuzzy object partitioning membership
***v***_***cj***_	Fuzzy feature partitioning membership
***d***_***ij***_	Relatedness measure between an object and a feature
***d***_***cij***_	Information bottleneck based distance between feature point *d*_*ij*_ and feature cluster centroid *p*_*cj*_
***p***_***cj***_	Feature cluster centroid
***T***_***u***_**, *T***_***v***_	Co-clustering user-defined parameters
***τ***	Number of iterations
***τ***_***max***_	Maximum number of iterations parameter
***ξ***	Convergence indicator

**Table 2:** Comparison of some popular fuzzy co-clustering algorithms.

Name	Objective function	Description
FCM	$J_{FCM}=\sum_{c=1}^{C}\sum_{i=1}^{N}u_{ci}^{m}{\left\|x_{i}-v_{c}\right\|}^{2}$	*m*≥1, *x*_*i*_ is the *i*-th object, *v*_*c*_ is centroid of the *c*-th cluster
FCCM	$\begin{array}{l} J_{FCCM}=\sum_{c=1}^{C}\sum_{i=1}^{N}\sum_{j=1}^{K}u_{ci}v_{cj}d_{ij}-T_{u}\sum_{c=1}^{C}\sum_{i=1}^{N}u_{ci}\ln u_{ci}\\ \;-T_{v}\sum_{c=1}^{C}\sum_{j=1}^{K}v_{cj}\ln v_{cj} \end{array}$	
FCR	$\begin{array}{l} J_{FCR}=\sum_{c=1}^{C}\sum_{i=1}^{N}\sum_{j=1}^{K}u_{ci}v_{cj}d_{ij}+R-T_{u}\sum_{c=1}^{C}\sum_{i=1}^{N}u_{ci}\ln u_{ci}\\ \;-T_{v}\sum_{c=1}^{C}\sum_{j=1}^{K}v_{cj}\ln v_{cj} \end{array}$	$\begin{array}{l} R=B\times \sum_{i=1}^{N}\sum_{j=1}^{K}d_{ij}\\ =\frac{NK-\sum_{d=1}^{C}\sum_{p=1}^{N}\sum_{q=1}^{K}u_{dp}v_{dp}}{NK}\times \sum_{i=1}^{N}\sum_{j=1}^{K}d_{ij} \end{array}$
FCCI	$\begin{array}{l} J_{FCCI}=\sum_{c=1}^{C}\sum_{i=1}^{N}\sum_{j=1}^{K}u_{ci}v_{cj}D_{cij}+T_{u}\sum_{c=1}^{C}\sum_{i=1}^{N}u_{ci}\ln u_{ci}\\ \;+T_{v}\sum_{c=1}^{C}\sum_{j=1}^{K}v_{cj}\ln v_{cj} \end{array}$	*D*_*cij*_ = (*d*_*ij*_−*p*_*cj*_)^2^
PFCC	$\begin{array}{l} J_{PFCC}=\sum_{c=1}^{C}\sum_{i=1}^{N}\sum_{j=1}^{K}\left(\frac{u_{ci}^{pos}}{\sum_{p=1}^{N}u_{cp}^{pos}}\right)\left(v_{cj}+w_{cj}\right)d_{ij}\\ \;-T_{u}\sum_{c=1}^{C}\sum_{i=1}^{N}{\left(u_{ci}^{pos}\right)}^{2}-T_{v}\sum_{c=1}^{C}\sum_{j=1}^{K}v_{cj}\ln v_{cj}\\ \;-T_{w}\sum_{c=1}^{C}\sum_{j=1}^{K}w_{cj}\ln w_{cj} \end{array}$	*upos ci* is the document possibilistic membership, *w*_*cj*_ is the word partitioning membership, *T*_*w*_ is a user-defined parameter
RFCC	$\begin{array}{l} J_{RFCC}=\sum_{c=1}^{C}\sum_{i=1}^{N}\sum_{j=1}^{K}\left(u_{ci}+x_{ci}\right)v_{cj}d_{ij}\\ \;-T_{u}\sum_{c=1}^{C}\sum_{i=1}^{N}u_{ci}\ln u_{ci}-T_{v}\sum_{c=1}^{C}\sum_{j=1}^{K}v_{cj}\ln v_{cj}\\ \;-T_{x}\sum_{c=1}^{C}\sum_{i=1}^{N}x_{ci}\ln x_{ci} \end{array}$	*x*_*ci*_ is a new additional and robust type of object membershp, *T*_*x*_ is a user-defined parameter
SS-HFCR	$\begin{array}{l} J_{SS-HFCR}=\sum_{c=1}^{C}\sum_{i=1}^{N}\sum_{j=1}^{K}u_{ci}v_{cj}d_{ij}-T_{u}\sum_{c=1}^{C}\sum_{i=1}^{N}u_{ci}\ln u_{ci}\\ \;-T_{v}\sum_{c=1}^{C}\sum_{j=1}^{K}v_{cj}\ln v_{cj}\\ \;+T_{u}\times T_{d}\left(\sum_{(x_{i},x_{k})\in ml}\sum_{c=1}^{C}u_{ci}u_{ck}-\sum_{(x_{i},x_{k})\in cnl}\sum_{c=1}^{C}u_{ci}u_{ck}\right) \end{array}$	*x*_*i*_ is the *i*-th object, *ml* and *cnl* are the training sets which contain *must*-*link* and *cannot*-*link* document pairs respectively, *T*_*d*_ is the weighting factor of a constraint

The FCCI algorithm is one of the most important fuzzy co-clustering algorithms. This algorithm includes a multi-dimensional distance function as the dissimilarity measure and entropy as the regularization term in its objective function. The FCCI emphasizes the importance of distance function, and its distance function equals the square of the *Euclidean* distance between feature data point and the feature cluster centroid. However, we all know that there are many similarity measures in the fields of data mining and pattern recognition[21]. The previous work of ours as well as other researchers’ show that information bottleneck based similarity measure is a more desirable choice because this similarity measure proves much better and can achieve much higher accuracy than other measures in clustering[22–24]. In the work of S. Noam and T. Naftali [23], the experimental results showed the average performance over all datasets attained 0.55 accuracy, while the second best result was 0.47 accuracy. Ye et al. [25] presented a novel alternative clustering algorithm, named SmIB, which employed mutual information to measure the information resided in data, and experimental results demonstrated that the SmIB algorithm was superior to the existing state-of-the-art alternative clustering algorithms.

Above analysis motivates us to present a novel *F*uzzy *C*o-*C*lustering algorithm based on *i*nformation *b*ottleneck similarity measure, called ibFCC. This approach assigns membership functions to both the objects and the features. Besides, because the biomedical data comes in a variety of forms, it is difficult for us to select just one appropriate method to calculate the pair-wise object similarity. We think the information bottleneck based similarity measure is much more appropriate. In the ibFCC, an objective function is formulated, which includes a distance function that employs information bottleneck theory to measure the similarity between feature data point and the feature cluster centroid.

The remainder of this paper is organized as follows. We firstly introduce in details the ibFCC, and then present our experimental results on five datasets, *Ohsumed* [26], *Lung Cancer* [27], *Breast Tissue* [28], *Cardiotocography* [28] and *Mice Protein Expression* [28]. Finally, we conclude our work.

## Methods

### The ibFCC algorithm

**Fig 1:**
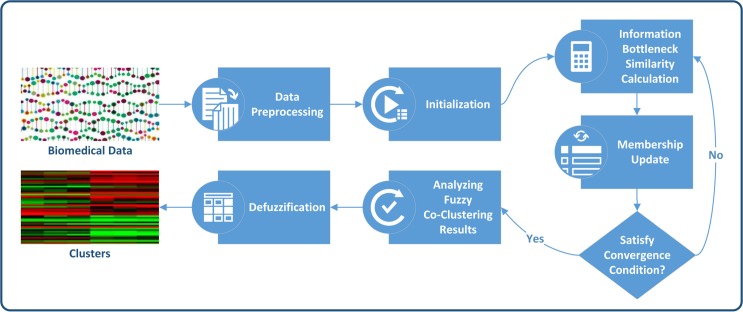
The flowchart of the proposed algorithm.

Since distance function is very necessary for fuzzy co-clustering to create richer co-clusters [17], FCCI includes the *Euclidean* distance function of feature data points from the feature cluster centroids in the co-clustering process. However, as we all know, there are so many other distance measures besides *Euclidean* distance function that it is difficult for users to choose an appropriate one. Too often this is an arbitrary choice. In the study of clustering, information bottleneck based distance measure proves much better. Therefore, the ibFCC algorithm we proposed employs information bottleneck theory to measure distance between feature data points and the feature cluster centroids. The overall clustering process is illustrated in Fig 1.

The goal of ibFCC is to minimize the objective function in Eq 1, subject to the following constraints in Eqs 2 and 3.

$$J_{ibFCC}=\sum_{c=1}^{C}\sum_{i=1}^{N}\sum_{j=1}^{K}u_{ci}v_{cj}d_{cij}+T_{u}\sum_{c=1}^{C}\sum_{i=1}^{N}u_{ci}\ln u_{ci}+T_{v}\sum_{c=1}^{C}\sum_{j=1}^{K}v_{cj}\ln v_{cj}$$ (1)

$$\sum_{c=1}^{C}u_{ci}=1,\;for\;i=1,2\cdots N$$ (2)

$$\sum_{j=1}^{K}v_{cj}=1,\;for\;c=1,2\cdots C$$ (3)

The first term in Eq 1 is the degree of aggregation that should be minimized during co-clustering, which intends to enable highly related *objects-features* to be co-clustered together. The *u*_*ci*_ and *v*_*cj*_ are two membership functions, indicating memberships of documents and features, respectively. The second and third terms are entropy regularization factors that combine all *u*_*ci*_'s and *v*_*cj*_'s separately. They control the degree of fuzziness in final clusters, where *T*_*u*_ and *T*_*v*_ are weighting parameters.

The constrained optimization of ibFCC can be solved by applying the Lagrange multipliers *α*, *β* to constraints in Eqs 2 and 3 respectively.

$$\begin{array}{l} J_{ibFCC}^{'}=\sum_{c=1}^{C}\sum_{i=1}^{N}\sum_{j=1}^{K}u_{ci}v_{cj}d_{cij}+T_{u}\sum_{c=1}^{C}\sum_{i=1}^{N}u_{ci}\ln u_{ci}+T_{v}\sum_{c=1}^{C}\sum_{j=1}^{K}v_{cj}\ln v_{cj}\\ \;+\sum_{i=1}^{N}\alpha_{i}(\sum_{c=1}^{C}u_{ci}-1)+\sum_{c=1}^{C}\beta_{c}(\sum_{j=1}^{K}v_{cj}-1) \end{array}$$ (4)

Take the partial derivative of *J*’ *ibFCC* in Eq 4 with respect to *U* and *V* respectively and set the gradient to zero, and then we have,

$$\frac{\partial J_{ibFCC}^{,}}{\partial U}=\sum_{j=1}^{K}v_{cj}d_{cij}+T_{u}(1+\ln u_{ci})+\alpha_{i}=0$$ (5)

$$\frac{\partial J_{ibFCC}^{,}}{\partial V}=\sum_{i=1}^{N}u_{ci}d_{cij}+T_{v}(1+\ln v_{cj})+\beta_{c}=0$$ (6)

Solving above equations yields the formulae for *u*_*ci*_, *v*_*cj*_ as:

$$u_{ci}=\frac{\exp\{-\sum_{j=1}^{K}v_{cj}d_{cij}/T_{u}\}}{\sum_{c=1}^{C}\exp\{-\sum_{j=1}^{K}v_{cj}d_{cij}/T_{u}\}}$$ (7)

$$v_{cj}=\frac{\exp\{-\sum_{i=1}^{N}u_{ci}d_{cij}/T_{v}\}}{\sum_{j=1}^{K}\exp\{-\sum_{i=1}^{N}u_{ci}d_{cij}/T_{v}\}}$$ (8)

Eqs 7 and 8 are the update equations for the document and feature memberships, where *d*_*cij*_ is distance between feature data point and the feature cluster centroid.

Let *c*_1_ and *c*_2_ be two clusters, and the distance between *c*_1_ and *c*_2_ is measured by information loss due to the merging of *c*_1_ and *c*_2_ based on Eq 9 as follows,

$$\begin{array}{l} d(c_{1},c_{2})=I(C_{before},Y)-I(C_{after},Y)\\ =\sum_{y,i\in \{1,2\}}p(c_{i},y)\log\frac{p(y|c_{i})}{p(y|c_{1}\cup c_{2})}\\ =\sum_{y,i\in \{1,2\}}p(c_{i})p(y|c_{i})\log\frac{p(y|c_{i})}{p(y|c_{1}\cup c_{2})}\\ =\sum_{i\in \{1,2\}}p(c_{i})\sum_{y}p(y|c_{i})\log\frac{p(y|c_{i})}{p(y|c_{1}\cup c_{2})} \end{array}$$ (9)

where *I*(*C*_*before*_, *Y*) and *I*(*C*_*after*_, *Y*) are the mutual information before and after the two clusters, *c*_1_ and *c*_2_, are merged together, *C*_*before*_ and *C*_*after*_ are the clusters before and after the mergence, *Y* is the feature space, and *y* is one feature.

Let the *i*^th^ document be a singleton cluster *sc*_*i*_, *x*_*ij*_ denotes the *j*^th^ feature value of the *i*^th^ document, *P* = {*p*_*cj*_} be the set of feature cluster centroids. Thus, Eq 9 can be rewritten to calculate the distance between this cluster *sc*_*i*_ and the *c*^*th*^ cluster, as

$$d(sc_{i},c)=\frac{\left|sc_{i}\right|}{N}\sum_{j=1}^{K}x_{ij}\log\frac{x_{ij}}{t_{cij}}+\frac{\left|c\right|}{N}\sum_{j=1}^{K}p_{cj}\log\frac{p_{cj}}{t_{cij}}$$ (10)

where |*sc*_*i*_| = 1 because this cluster has only one object. The *d*_*cij*_ is the *j*-th component product of *d*(*sc*_*i*_, *c*), and we can get,

$$d_{cij}=\frac{1}{N}x_{ij}\log(x_{ij}/t_{cij})+\frac{\left|c\right|}{N}p_{cj}\log(p_{cj}/t_{cij})$$ (11)

where *t*_*cij*_ = (*x*_*ij*_+|*c*|*p_*cj*_)/(1+|*c*|), |*c*| is the number of documents in the *c*^th^ cluster. It is a little more complicated to define the value of |*c*| in fuzzy clustering than in hard clustering, because we need to perform defuzzification operation on the fuzzy membership matrix. After defuzzification, we can get the value of |*c*| as easily as in hard clustering.

Note that in our ibFCC, it is difficult to get the value of *p*_*cj*_ explicitly. Even if the value of *p*_*cj*_ may be calculated as *u*_*ci*_ and *v*_*cj*_ theoretically, the process may suffer from high computational complexity mathematically. Thus we choose an alternative approach which employs a weighted averaging method. In fuzzy clustering, the centroid of a cluster is the mean of all points, weighted by their degree of belonging to the cluster. And then we have the normalized update equation of *p*_*cj*_,

$$p_{cj}=\frac{\sum_{i=1}^{N}u_{ci}x_{ij}}{\sum_{i=1}^{N}u_{ci}}$$ (12)

Through Eqs 7 and 8, the solution of the constrained optimization problem in Eq 4 can be approximated by Picard iteration. The proof of convergence of the ibFCC algorithm is given in the Appendix section of this paper. The pseudocode of ibFCC is given in Algorithm 1.

**Algorithm 1. ibFCC algorithm**.

**1:** Set values of parameters *C*, *T*_*u*_, *T*_*v*_ maximum error limit *ξ* and the maximum number of iterations parameter *τ*_*max*_

**2:** Set *τ* = 1

**3:** Initialize memberships *u*_*ci*_ and *v*_*cj*_ randomly

**4:** **REPEAT**

**5:**    Calculate the value of *p*_*cj*_ using Eq 12

**6:**    Calculate the information bottleneck distance *d*_*cij*_ using Eq 11

**7:**    Update membership *v*_*cj*_ using Eq 8

**8:**    Update membership *u*_*ci*_ using Eq 7

**9:**    Set *τ* ++

**10: UNTIL** *max*(|*u*_*ci*_(*τ*)-*u*_*ci*_(*τ*-1)|)≤*ξ* or *τ* = *τ*_*max*_

The pseudo-code of ibFCC shows that the time complexity of ibFCC is *O*(*CNKτ*), where *τ* denotes the number of iterations. Its time complexity is equivalent to such fuzzy co-clustering algorithms as FCCM and FCCI with *O*(*CNKτ*).

### Algorithm effectiveness tests

In order to test the effectiveness of ibFCC, we carried out a set of experiments. The experimental results are also compared with four well received approaches in the literature, FCM, FCCM, RFCC and FCCI. Of the four algorithms, FCM is a standard fuzzy clustering algorithm, and the others are fuzzy co-clustering algorithms.

#### Experimental setup

We employed five datasets to evaluate the performance of ibFCC in categorizing real-world data, *Ohsumed*, *Lung Cancer*, *Breast Tissue*, *Cardiotocography* and *Mice Protein Expression*.

1) The *Ohsumed* corpus is the collection consisting of the first 20,000 documents from the 50,216 medical abstracts of the year 1991. The classification scheme consists of the 23 Medical Subject Headings (MeSH) diseases categories. Based on the *Ohsumed* corpus, we constructed two subsets, *Oh*1 and *Oh*2, which are introduced in **Table 3**. In our experiments on the *Ohsumed* corpora, we selected top 500 features, that is, *K* = 500.

**Table 3:** Dataset details.

Dataset	#Categories	#Samples	#Features
***Oh*1**	5	1000	500
***Oh*2**	10	1000	500
***LC***	3	27	56
***BT***	6	106	9
***Card***	10	2126	21
***MPE***	8	1076	68

2) The *Lung Cancer* (*LC*) dataset is used by Hong and Young to illustrate the power of the optimal discriminant plane even in ill-posed settings. It contains 27 instances and 56 attributes. We used the existing classification as our baseline on how the dataset should be clustered.

3) The *Breast Tissue* (*BT*) corpus can be used for predicting the classification of either the original 6 classes or of 4 classes by merging together the fibro-adenoma, mastopathy and glandular classes whose discrimination is not important (they cannot be accurately discriminated anyway). It contains 106 instances and 9 attributes.

4) In the *Cardiotocography* (*Card*) dataset, 2126 fetal cardiotocograms (CTGs) were automatically processed and the respective diagnostic features were measured. The CTGs were also classified by three expert obstetricians with a consensus classification label assigned to each of them.

5) This *Mice Protein Expression* (*MPE*) dataset contains a total of 1076 measurements per protein. Each measurement can be considered as an independent sample/mouse. The eight classes of mice are described based on features such as genotype, behavior and treatment.

#### Evaluation criteria

There are several ways for numerically scoring the cluster quality, such as Entropy, F-Measure and Overall Similarity. We choose F-Measure, Entropy and p-value as the criteria to evaluate the performance of ibFCC.

F-Measure is the weighted harmonic mean of precision and recall. In terms of evaluating clustering accuracy, the higher the value of F-Measure is, the better the clustering quality is. And the F-Measure value of is given by:

$$F(i,j)=\frac{2*\;precision(i,j)*\;recall(i,j)}{precision(i,j)+recall(i,j)}$$ (13)

where *precision*(*i*,*j*) and *recall*(*i*,*j*) are computed using the following equations respectively:

$$recall(i,j)=n_{ij}/n_{i}$$ (14)

$$precision(i,j)=n_{ij}/n_{j}$$ (15)

where *n*_*ij*_ is the number of members of class *i* in cluster *j*, *n*_*j*_ is the number of members of cluster *j*, and *n*_*i*_ is the number of members of class *i*. The overall value for the F-Measure is given by the following:

$$F_{c}=\sum_{i}\frac{n_{i}}{n}\;*\;\max\{F(i,j)\}$$ (16)

where *n* is the total number of documents.

The Entropy can also be used to evaluate cluster distribution during clustering in information theory. The expression for Entropy of clustering result is listed as follows:

$$E_{cs}=\sum_{j=1}^{m}\frac{n_{j}E_{j}}{n}$$ (17)

where *E*_*cs*_ is the whole Entropy value, *n*_*j*_ is the number of documents in cluster *j*, *n* is the number of all the documents, *m* is the number of clusters and *E*_*j*_ is the Entropy value of cluster *j*, which is calculated using the following formula:

$$E_{j}=-\sum_{i}^{}p_{ij}\;\log\;p_{ij}$$ (18)

where *p*_*ij*_ is the probability that one document belonging to class *i* could be put into cluster *j* during the partition. It should be noted that the lower the value of Entropy, the higher the clustering quality will be.

In research of GO (Gene Ontology) whose objective is to provide controlled vocabularies for the description of the biological process, molecular function, and cellular component of gene products, the p-value is often used to calculate the statistical significance of a group of proteins that shares a GO term [29]. In the dataset, given *N* proteins where *M* of them have the same annotation, the probability of observing *m* or more proteins that are annotated with the same GO term out of *n* proteins is,

$$p-value=\sum_{i=m}^{n}\frac{\left({}_{\;i}^{M}\right)\left({}_{\;n-i}^{N-M}\right)}{\left({}_{n}^{N}\right)}$$ (19)

A cluster with a smaller p-value is usually more significant than one with a higher p-value. After getting the p-value of each single cluster, the quality of overall clusters could be measured by the CS (clustering score) function, which is calculated as follows.

$$CS=\frac{\sum_{i=1}^{ns}min\left(p_{i}\right)+\left(nl*cutoff\right)}{ns+nl}$$ (20)

where *ns* and *nl* is the number of significant and insignificant clusters, respectively. The *cutoff* denotes the *α* level (0.05), and if a group of proteins are associated with a p-value less than the cutoff, they are considered significant, and vice versa. The *min*(*p*_*i*_) is the smallest p-value of the significant cluster *i*.

## Results

We firstly compared the performances of FCM, FCCM, RFCC, FCCI and ibFCC on the six subsets. All the five algorithms were initialized randomly and run for ten times to reduce the impact of local optimizations.

**Fig 2:**
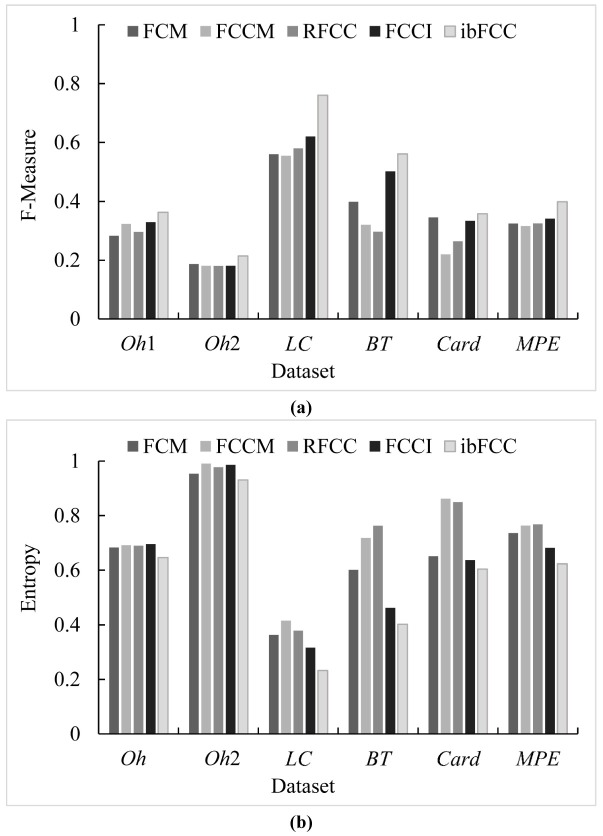
Clustering performance comparisons of FCM, FCCM, RFCC, FCCI and ibFCC in terms of F-Measure and entropy on the six subsets. (a) F-Measure values of FCM, FCCM, RFCC, FCCI and ibFCC (b) Entropy values of FCM, FCCM, RFCC, FCCI and ibFCC.

The clustering performance comparisons of the five algorithms are illustrated in Fig 2. On the six subsets (*Oh*1, *Oh*2, *LC*, *BT*, *Card*, *MPE*), the accuracy of ibFCC is much better, which is calculated to be 0.36, 0.21, 0.76, 0.56, 0.36 and 0.40 respectively in terms of F-Measure, and 0.65, 0.93, 0.23, 0.40, 0.60 and 0.62 respectively in terms of Entropy. Besides ibFCC, FCCI values 0.33, 0.18, 0.62, 0.50, 0.33 and 0.34 in terms of F-Measure, and 0.70, 0.99, 0.32, 0.46, 0.64 and 0.68 in terms of Entropy, whose performance is relatively better than FCM, FCCM and RFCC. At the same time, we observed that the F-Measure values of these algorithms are higher, and the Entropy values are lower, when the value of *C* is small. As the value of *C* increases, the F-Measure and Entropy values show that the performances of these clustering algorithms reduce, however, the clustering accuracy of the ibFCC is still the highest.

**Fig 3:**
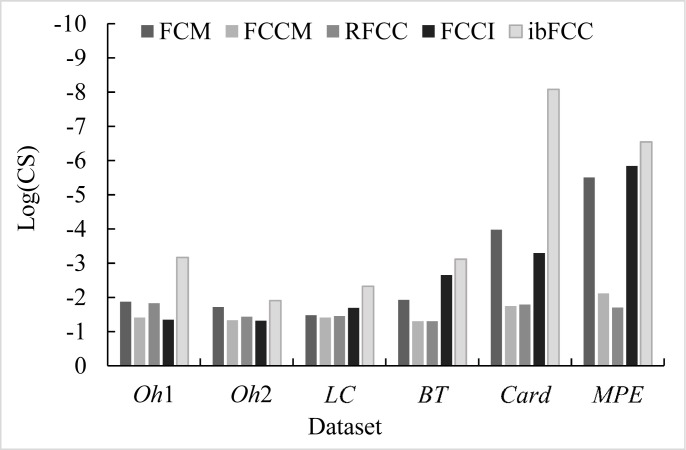
Clustering performance comparisons of FCM, FCCM, RFCC, FCCI and ibFCC in terms of clustering score on the six subsets.

In addition to F-Measure and Entropy, we chose clustering score and p-value to further evaluate the performances of the ibFCC. The experimental results in terms of clustering score are illustrated as Fig 3, which shows the comparison of the five algorithms. On the six subsets, the clustering score values of the ibFCC are much lower, and thus this algorithm achieves a significant improvement than the counterparts. However, on the *BT* and *MPE* dataset, the clustering score value of ibFCC is only slightly less than FCCI, which shows that the clustering accuracy of these two algorithm is similar. To be sure, the experimental results illustrated in Fig 2 are average values of 10-times clustering experiments, but Fig 3 only lists the results of a single clustering experiment, in order to calculate the values of clustering score and p-value. And therefore, in Fig 3, stochastic fluctuation of the experimental results is strong. In addition, in clustering results of FCCI and FCM, there are often some empty clusters, which will easily bring a higher clustering accuracy (higher F-Measure value and lower Entropy value) because the number of *C* is lower.

**Fig 4:**
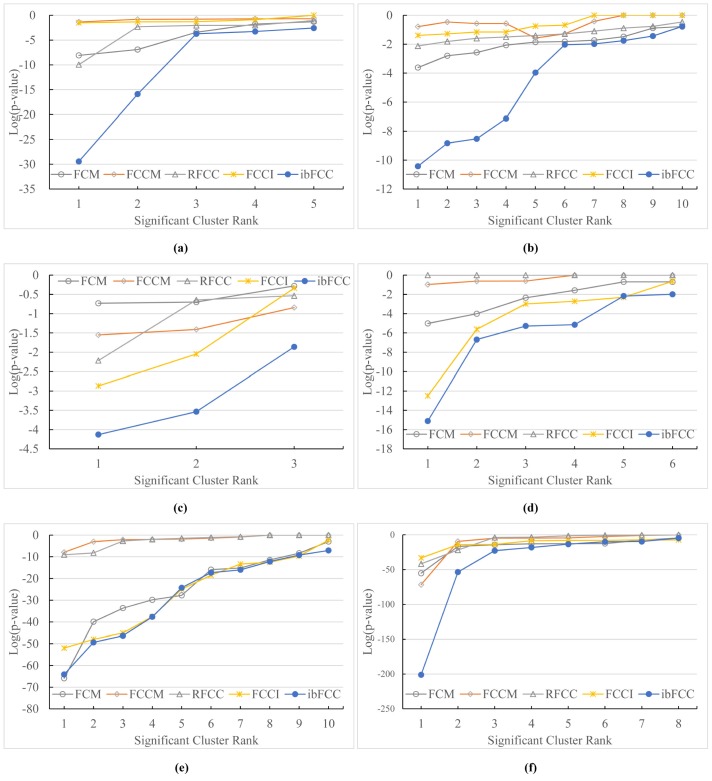
Comparisons of the five approaches on the 6 subsets in terms of p-value. (a) *Oh*1(b) *Oh*2(c) *LC*(d) *BT*(e) *Card*(f) *MPE*.

The following experiments illustrate the significance of our clustering results in terms of p-value. Experimental results on the six subsets are listed as Fig 4A, 4B, 4C, 4D, 4E and 4F, respectively. In Fig 4A, the p-values of the best clusters of the five algorithms are 8.0E-09, 0.045, 1.1E-10, 0.031 and 3.6E-30, respectively. And similarly, our algorithm has or approaches (only on the *Card* subset in Fig 4E) the lowest p-value. Results of this set of experiments show that biomedical data can be grouped into more meaningful clusters, and our algorithm could provide more significant clusters.

**Fig 5:**
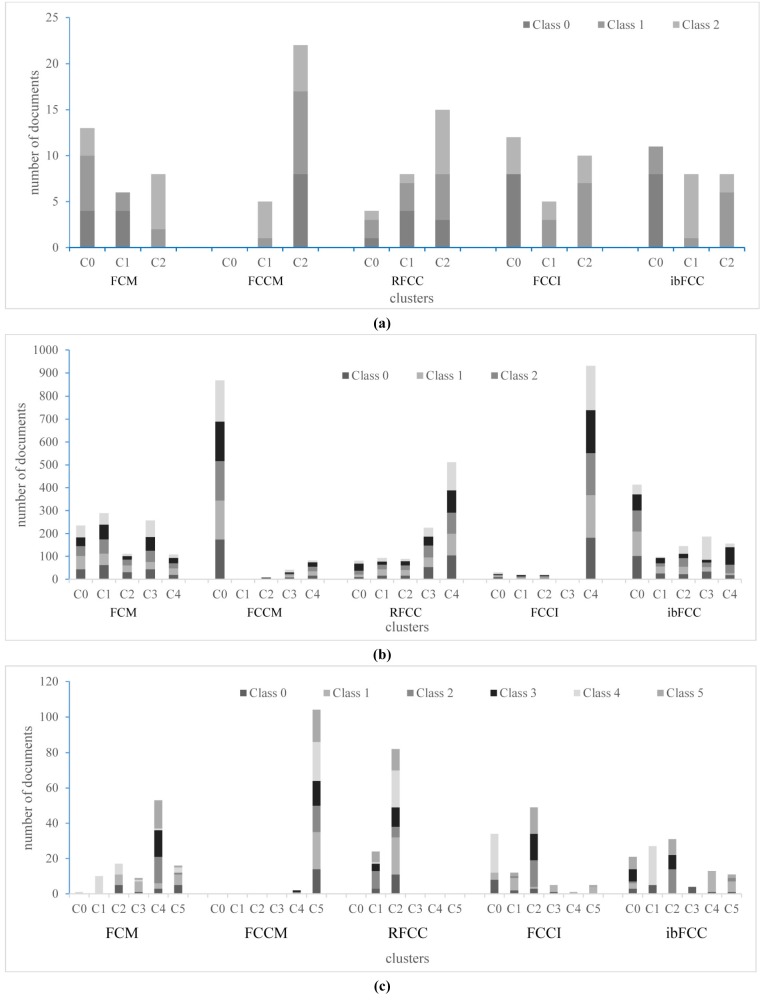
Document clusters distribution for three subsets with less clusters. (a) *LC*(b) *Oh*1(c) *BT*.

The corresponding document cluster distributions are shown in Fig 5. Clustering results of *LC*, *Oh*1 and *BT* are illustrated as Fig 5A, 5B and 5C. Because the number of clusters is large on *Oh*2, *Card* and *MPE* datasets, it is difficult for clustering results to be illustrated in figures. And the experiments on the *Oh*2, *Card* and *MPE* datasets are not discussed here. It can be seen from Fig 5 that ibFCC can generate clusters better than other algorithms. Fig 5 shows that ibFCC well generates C1 on *LC*, C3 and C4 on *Oh*1, C1, C3 and C4 on *BT*. Clustering performances of FCCM and RFCC are similar, and it is difficult for these two algorithms to capture categories properly. FCM and FCCI perform well on a part of datasets such as the *LC* subset.

## Discussion

In our experiments, some clusters have few documents, such as some clusters generated by FCCI. We gave some analysis on the problem and concluded that when datasets were sparse and high-dimensional, all the objects could be assigned to a single cluster in FCM-type clustering [30]. The six subsets are exactly sparse, and thus in clustering results of such fuzzy co-clustering algorithms as FCCM, RFCC and FCCI, some clusters have no objects (as Fig 5), which will significantly reduce clustering performance.

To avoid the problem, Mei et al. [30] proposed a method to normalize all the centroids to unit norm after each iteration

$$\delta_{c}^{'}=\delta_{c}/\|\delta_{c}\|$$ (21)

where *δ*_*c*_ is the centroid of the *c*-th cluster, and

$$\|\delta_{c}\|=\sqrt{{\sum_{j}\left(\sum_{i}u_{ci}^{m}w_{i}d_{ij}\right)}^{2}}/\sum_{i}u_{ci}^{m}w_{i}$$ (22)

where *m* is a constant, *w*_*i*_ controls the weights of objects, and *δ*’ *c* is the normalized centroid. Their algorithm is an incremental clustering method, and thus does not appear in our experiments.

**Fig 6:**
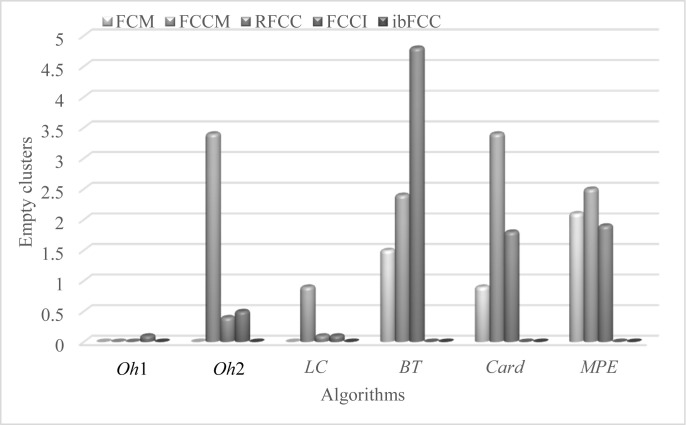
Comparison of our approaches and counterpart algorithms in terms of empty cluster number.

In ibFCC, centroids and objects are assigned different weights in calculating information bottleneck based similarity, as Eq 11, which is equivalent to the normalization process of Mei et al. Therefore, in experimental results of ibFCC, there are less empty clusters, and clustering performance is much better. Fig 6 illustrates the average number of empty clusters in our experiments on the six subsets. In Fig 6, there are some empty clusters in the results of FCM, FCCM and RFCC. The average numbers of empty clusters of the RFCC are 0.4, 0.1, 4.8, 1.8 and 1.9 on the *Oh*2, *LC*, *BT*, *Card* and *MPE*, respectively. If there are more than one empty clusters in clustering results, the clustering quality will be significantly reduced, although the value of F-Measure is higher and the value of Entropy is lower. The ibFCC generates almost zero empty clusters in the results, and therefore, this algorithm outperforms the counterparts. The FCCI algorithm has the second best clustering results, with only 0.1, 0.5, 0.1 empty clusters on *Oh*1, *Oh*2 and *LC* subsets respectively.

In addition to the number of empty result clusters, running time is also an important issue. As indicated earlier, the time complexity of ibFCC is *O*(*CNKτ*), which is equivalent to such fuzzy co-clustering algorithms as FCCM, RFCC and FCCI. Even if the FCM algorithm implements fuzzy clustering rather than fuzzy co-clustering, its time complexity is also *O*(*CNKτ*). However, time complexity merely manifests the conclusion of theoretical analysis. In order to thoroughly compare these algorithms, we carried out additional experiments to record clustering time. The running time required by every algorithms to complete oncethrough clustering on each dataset is listed as Table 4. The comparison indicates that, on the six datasets, the FCM algorithm is the most time-consuming. The main reason lies in that this algorithm is sensitive to noise, which reduces significantly the convergence speed. Although other four fuzzy co-clustering algorithms seem to be more complex, they group objects as well as features, which could help to significantly reduce feature dimension and improve clustering efficiency. Thus it can be justified again that the fuzzy co-clustering algorithms are better than fuzzy clustering algorithms. The comparisons of the four fuzzy co-clustering algorithms in terms of running time show that the FCCM performs the best, and the ibFCC takes longer time. The former is because computational procedure of the FCCM is very easy, and the latter is because of the complex similarity measure based on information bottleneck of ibFCC. The similarity measure of FCCI is more complex than FCCM and RFCC, which makes FCCI needs more time to complete clustering. Similarly, the information bottleneck based measure of ibFCC is more time-consuming than FCCI, and therefore, the running time of ibFCC is longer than FCCI. Even so, the ibFCC is still more efficient than FCM. In conclusion, the ibFCC achieves high clustering accuracy while encountering more actual running time because of the calculation process of similarity measure, although its theoretical time complexity does not increase. Therefore, it will be a study emphasis in our further research how to further improve actual running efficiency.

**Table 4:** Comparison of our approach and counterpart algorithms in terms of running time (*s*).

Datasets	FCM	FCCM	RFCC	FCCI	ibFCC
***Oh*1**	1.606	0.115	0.210	0.412	0.863
***Oh*2**	3.203	0.223	0.363	0.730	1.670
***LC***	0.007	0.002	0.002	0.002	0.007
***BT***	0.007	0.002	0.001	0.003	0.007
***Card***	0.289	0.016	0.029	0.061	0.216
***MPE***	0.573	0.015	0.048	0.105	0.419

## Conclusion

Recently, several fuzzy co-clustering algorithms have been proposed. Keeping the advantages of co-clustering and fuzzy clustering, these algorithms improve the representation of overlapping clusters by using fuzzy membership function, and greatly facilitate the reorganization of large biomedical data.

In existing prominent fuzzy co-clustering algorithms, *Euclidean* distance function is the most frequently used. However, information bottleneck based distance measure proves much better in many clustering algorithms. Therefore, in this paper we propose a novel fuzzy co-clustering algorithm, named ibFCC, whose objective function includes an information bottleneck based distance function to measure distance between feature data points and the feature cluster centroids. We implement experiments on five biomedical datasets, *Ohsumed*, *Lung Cancer*, *Breast Tissue*, *Cardiotocography* and *Mice Protein Expression*, to evaluate the performance of ibFCC. Our algorithm is also compared with some popular fuzzy (co-)clustering algorithms and proves to outperform them.

It is challenging to determine the number of clusters in the literature. In our study, the value of *C* is still specified by users manually, which determines that ibFCC is not unsupervised absolutely. In the future, we intend to incorporate techniques evaluating the number of clusters to optimize our approach.

## Appendix

The proof of convergence of our algorithm is shown below:

Based on the bounded monotonic principle, we know that a monotone bounded function is convergent. Therefore, in order to prove the convergence of ibFCC, we need to prove that the value of *J*_ibFCC_ never increases when we update Eqs 12, 11, 8 and 7, and *J*_ibFCC_ is a bounded function.

### Theorem 1

In every iteration, the updated value of *u*_*ci*_ given by Eq 7 never increases the value of the objective function *J*_ibFCC_ in Eq 1.

### Proof

We consider the objective function of *J*_ibFCC_ as a function of a single variable *u*_*ci*_, denoted by *J*(*U*):

$$J\left(U\right)=\sum_{c=1}^{C}\sum_{i=1}^{N}\sum_{j=1}^{K}u_{ci}v_{cj}d_{cij}+T_{u}\sum_{c=1}^{C}\sum_{i=1}^{N}u_{c}{}_{i}\ln u_{c}{}_{i}+constant$$ (23)

where

$$constant=T_{v}\sum_{c=1}^{C}\sum_{j=1}^{K}v_{cj}\ln v_{cj}$$ (24)

Similarly, the variables *v*_*cj*_ and *d*_*cij*_ may be considered as two constants. And then theorem 1 can be proven by showing that the u* (i.e., the updated value of *u*_*ci*_ given by Eq 7) is the local minima of the objective function *J*(*U*) by Lagrange multiplier method. For this we need to prove that the Hessian matrix △^2^*J*(u^*^) is positive definite.

$$\Delta^{2}J(u)=\left[\begin{array}{ccc} \frac{\partial^{2}J(u)}{\partial u_{11}\partial u_{11}} & \cdots & \frac{\partial^{2}J(u)}{\partial u_{11}\partial u_{CN}}\\ \vdots & \ddots & \vdots \\ \frac{\partial^{2}J(u)}{\partial u_{CN}\partial u_{11}} & \cdots & \frac{\partial^{2}J(u)}{\partial u_{CN}\partial u_{CN}} \end{array}\right]=\left[\begin{array}{ccc} \frac{T_{u}}{u_{11}} & \cdots & 0\\ \vdots & \ddots & \vdots \\ 0 & \cdots & \frac{T_{u}}{u_{CN}} \end{array}\right]$$ (25)

At u^*^, *u*_*ci*_≥0 and *T*_*u*_ is always assigned with a positive value. Therefore the Hessian matrix △^2^*J*(u^*^) is positive definite. In summary, u* is the objective function of stationary point ((∂*J* (*u*_*ci*_)/∂*u*_*ci*_) = 0) and Hessian matrix △^2^*J*(u^*^) is positive definite. By sufficient and necessary condition for the existence of extreme value of multivariate function knows that the updated *u*_*ci*_ is indeed a local minima of *J*(*U*) and it never increases the objective function value.

### Theorem 2

At every iteration, the updated values of *v*_*cj*_ given by Eq 8 never increase the objective function *J*_ibFCC_ in Eq 1.

### Proof

Theorem 2 can be proven in a similar fashion as Theorem 1.

### Theorem 3

The objective function of *J*_ibFCC_ in Eq 1 is bounded. In other words, there is a constant *M*, which makes the *J*_ibFCC_ more than *M* all the way (i.e., *J*_ibFCC_≥M).

### Proof

Since the minimum value of *u*_*ci*_ and *v*_*cj*_ is 0, and *d*_*cij*_≥0, we know that the first term of *J*_ibFCC_ is greater than or equal to 0, that is,

$$\sum_{c=1}^{C}\sum_{i=1}^{N}\sum_{j=1}^{K}u_{ci}v_{cj}d_{cij}*\geq 0$$ (26)

The second and third terms of *J*_ibFCC_ in Eq 1 are all entropy regularization terms, and when *u*_*ci*_ = 1/*C*, and *v*_*cj*_ = 1/*K*, the minimum value of the function will be achieved.

$$J_{ibFCC}\geq T_{u}*N*\log\frac{1}{C}+T_{v}*C*\log\frac{1}{K}$$ (27)

Because *T*_*u*_, *N*, *C*, *T*_*v*_ and *K* are all constants, we can get that *J*_ibFCC_≥*M*, when *M* = *T*_u_*N*log(1/*C*)+*T*_v_*C*log(1/*K*). In summary, the objective function *J*_ibFCC_ is bounded.

### Corollary 1

The ibFCC algorithm converges to a local minimum of the optimization, with the update formulae given in Eqs 12, 11, 8 and 7.

### Proof

This corollary is a direct consequence of the above three theorems. Theorems 1 and 2 indicate that the procedure of membership updating never increases the value of the ibFCC objective function. Theorem 3 states that there is a limit to how much this objective function can be decreased. So eventually the procedure should stop somewhere before or when it reaches this limit.
